# Computational Approach for Detection of Diabetes from Ocular Scans

**DOI:** 10.1155/2022/5066147

**Published:** 2022-05-14

**Authors:** Asif Irshad Khan, Pravin R. Kshirsagar, Hariprasath Manoharan, Fawaz Alsolami, Abdulmohsen Almalawi, Yoosef B. Abushark, Mottahir Alam, Fekadu Ashine Chamato

**Affiliations:** ^1^Computer Science Department, Faculty of Computing and Information Technology, King Abdulaziz University, Jeddah 21589, Saudi Arabia; ^2^Department of Artificial Intelligence G H Raisoni College of Engineering, Nagpur, Maharashtra, India; ^3^Department of Electronics and Communication Engineering, Panimalar Institute of Technology, Chennai, India; ^4^Department of Electrical and Computer Engineering, Faculty of Engineering, King Abdulaziz University, Jeddah 21589, Saudi Arabia; ^5^Department of Chemical Engineering, College of Biological and Chemical Engineering, Addis Ababa Science and Technology University, Addis Ababa, Ethiopia

## Abstract

The estimated 30 million children and adults are suffering with diabetes across the world. A person with diabetes can recognize several symptoms, and it can also be tested using retina image as diabetes also affects the human eye. The doctor is usually able to detect retinal changes quickly and can help prevent vision loss. Therefore, regular eye examinations are very important. Diabetes is a chronic disease that affects various parts of the human body including the retina. It can also be considered as major cause for blindness in developed countries. This paper deals with classification of retinal image into diabetes or not with the help of deep learning algorithms and architecture. Hence, deep learning is beneficial for classification of medical images specifically such a complex image of human retina. A large number of image data are considered throughout the project on which classification is performed by using binary classifier. On applying certain deep learning algorithms, model results into the training accuracy of 96.68% and validation accuracy of 66.82%. Diabetic retinopathy can be considered as an effective and efficient method for diabetes detection.

## 1. Introduction

Diabetes can be considered as one of the leading causes of visual impairment across the world. Significant progress has been made in understanding and treating diabetes, but population growth requires new management strategies in the future. Diabetic retinopathy (DR) is an ocular manifestation of physical injury at the end of diabetes. Diabetes is the most common disease across the world. Under the age group of 50 years, it serves as the most common cause of blindness. Over 10 years of timespan, it affected around 80% people. Most researchers agree that up to 90 percent of diabetic patients can be cured by early diagnosis. A person with diabetes is more likely to be at risk for diabetes retinopathy (DR) [1]. Blood flow to all layers of the retina is done through small blood vessels that are affected by uncontrolled blood sugar levels. When large amounts of glucose or fructose accumulate in the blood, vessels begin to break down due to insufficient oxygen supply to the cells. Any blockade on these vessels leads to serious eye damage. As a result, metabolic rate decreases and leads to structural abnormalities in vessels including diabetic retinopathy [2]. Diabetic retinopathy is a progressive disease, and early detection is very important in saving a patient's vision which requires constant eye check-ups. The diabetic retinopathy automated diagnosis program can help reduce the chances of complete blindness due to DR and reduce the operational burden on eye specialists. Examination of the severity and degree of retinopathy associated with a person with diabetes is currently being performed by medical professionals based on the fundus or in the patient's upper eyelids [3]. Therefore, the current study focuses on the application of a variety of automated learning techniques that focuses on effective stage analysis in the development of diabetes detection [4]. As the number of diabetic patients increases rapidly, the number of retinal images produced by screening programs will also increase, which in turn introduces a greater burden on medical professionals and the cost of health services.

**Figure 1:**
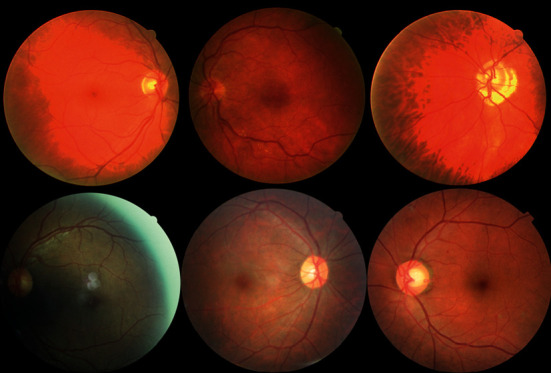
Overview of retinal images.

Diabetic retinopathy is best diagnosed with a comprehensive dilated eye exam. So, to detect it, Drops are placed in your eyes, which widen (dilate) your pupils to allow your doctor to better view inside your eyes. There are various symptoms of diabetic retinopathy such as spots or dark strings floating in your vision (floaters), blurred vision, fluctuating vision, impaired colour vision, dark or empty areas in your vision, and vision loss. There are basically three types of diabetic retinopathy, background retinopathy, preproliferative diabetic retinopathy, and proliferative diabetic retinopathy. For this task, we are making use of artificial intelligence which will give truthful, efficient, and effective results. Artificial intelligence contains various parts such as machine learning, deep learning, natural language processing, and computer vision. Throughout the proposed solution, we have used deep learning classification techniques with the help of its various algorithms and architectures as deep learning is very effective for classification tasks and was chosen for retinal image classification throughout the proposed task. Use of artificial intelligence in medicine in an evolving technology holds promise for mass screening and perhaps may even help in establishing an accurate diagnosis [5]. The ability of complex computing is to perform pattern recognition by creating complex relationships based on input data, and then comparing it with performance standards is a big step. Few glimpses of retinal fundus image are shown in Figure 1.

Diabetic retinopathy is an ever-increasing problem. Early screening and timely treatment of the same can reduce the burden of sight threatening retinopathy. So, we are aiming to build a tool which can aid in quick screening of this disorder, and minimal requirement of trained human resource for the same would probably be a boon for patients and ophthalmologists. Recent deep learning methods provide an effective way to construct an end-to-end model that can compute final classification labels with the raw pixels of medical images. Both quantitative performance and qualitative performance of this method were evaluated on three publicly available DRIVE, REVIEW, and STARE datasets [6]. Multidirectional morphological top-hat transform with rotating structuring elements and enhanced multiscale line detector is utilized for blood vessels detection [7]. Morphological operators are used for detecting blood vessel tree. In retinal fundus images, identification of abnormal spots is done more accurately after vessel detection.

### 1.1. Relevant Approach

A lot of work has been performed in this field, and there are various ways to get diabetic retinopathy. The discovery researchers have worked on various techniques such as finding blood vessels. The change in the shape and size of the blood vessels is a good indication for getting diabetic retinopathy. In the same way, the presence of various lesions helps in the diagnosis of diabetic retinopathy. Thus, various studies have been categorized into two mechanisms as blood vessels segmentation [8] and of identifying the lesions [9].  Table 1 shows the approach used by various researchers for diabetic retinopathy.

Therefore, from Table 1, it can be concluded that there is improvement in results regarding diabetic retinopathy by current techniques. Hence, deep learning is beneficial for classification of medical images specifically such a complex image of human retina.

## 2. System Model: Methodology and Dataset

The data play a vital role throughout. For the noble task, we have collected dataset from Kaggle which has the size of 271.39 MB and was divided into 5 classes, with images ranging from 0 to 4 according to the severity of the disease. It is large set of high-resolution retina images taken under a variety of imaging conditions as mentioned: 0 means No DR, 1 means mild, 2 means moderate, 3 means severe, and 4 means proliferative DR. A left and right field is provided for every subject. Images are labelled with a subject id as well as either left or right (e.g., 1_left.jpeg is the left eye of patient id 1). The images in the dataset come from different models and types of cameras, which can affect the visual appearance of left vs. right. Further, we made three folders for train, validation, and test purpose. Each folder contains malicious and normal folder containing images of retina images. The number of images are malicious and normal folder where same in for same directory. The train folder was used to train the deep learning model to detect the pattern in the image inside it. The validation folder was used to validate the data over it. The test was finally used to check the accuracy of the model over its images. Our modified data with detailed description of per folder image size are mentioned in Table 2. The images are equally divided for malicious and normal category for all training, testing, and validation.

The data set that is present in Table 2 must be normalized with respect to varying parameters as 141 image sets are represented. Therefore, normalization of images can be represented using the following equation:

(1) $$\begin{array}{c} \vartheta_{n}=\sum_{i=1}^{n}\frac{\vartheta_{o}-\vartheta_{s}}{\vartheta_{l}-\vartheta_{s}}, \end{array}$$

where *ϑ*_*o*_ indicates the reference values and *ϑ*_*l*_ and *ϑ*_*s*_ denote the boundary conditions of data set.

From (1), it can be computed that reference values will be directly provided as input in the training model and corresponding validation set is obtained with comparison values. Thus, the calculated mathematical solutions are represented in Table 2 where after certain comparison, it is observed that both malicious and normal images are equivalent at last stage due to exact comparison that is made using deep learning. In addition, the boundary values are defined in an exact way to avoid intersection points in the image processing technique. The normalized parameter can be updated using the following equation:

(2) $$\begin{array}{c} \vartheta_{n}\left(i,t\right)=\sum_{i=1}^{n}\left[{\text{decay}}_{i}\right]*\vartheta_{\text{in}}, \end{array}$$

where *ϑ*_*n*_(*i*, *t*) denotes the updated normalized values that vary with varying time and [decay_*i*_] represents the decaying values in matrix form.

From (2), it can be implicit that decaying values are added with respect to defined time values. However, varying time periods are measured with the equivalent diagonal matrix thus multiplying with normalized parameters. Conversely, information gain of normalized parameters must be measured to prove the effectiveness of the proposed model using deep learning. Thus, the information gain can be designed using the following equation:

(3) $$\begin{array}{c} G_{i}=\sum_{i=1}^{n}\tau \left|d_{i}\right|+\tau \left|AD_{i}\right|, \end{array}$$

where *τ* represents the information of comprehensive data set and *d*_*i*_ and *AD*_*i*_ denote observed data and adversarial data.

Equation (3) is applied before preprocessing stage as information gain must be compared after preprocessing steps. This kind of difference is made to achieve more than 50 percentage of effective transmissions during period cycle of deep learning procedures. Thus, the information gain after the preprocessing stage can be mathematically represented using the following equation:

(4) $$\begin{array}{c} \tau \left|d_{i}\right|=\sum_{i=1}^{n}\text{log}\left(\frac{AD_{i}}{d_{i}}\right)*\text{entropy}\left(d_{i}\right). \end{array}$$

From (4), both logarithmic values and entropy of defined data set are reserved in a direct form from all predefined data set. The distorted data set can be represented using the following equation:

(5) $$\begin{array}{c} \rho d_{i}=\sum_{i=1}^{n}\frac{{\text{input}}_{in}-\alpha }{\beta_{i}}, \end{array}$$

where input_in_ denotes the input parameters in distorted form and *α* and *β*_*i*_ represent the mean and standard values of processed images.

From (5), standard attribute values are obtained within the binary boundary limits [−1, 0, 1] thus achieving a proper data structure scheme even at crucial stages. The control parameter at crucial image transforming process can be represented using the following equation:

(6) $$\begin{array}{c} \delta_{i}=\sum_{i=1}^{n}w_{i}*\nabla_{in}\theta , \end{array}$$

where *w*_*i*_ denotes the weight of *i*^*th*^ loop.

From (6), continuous loop expression can be solved with different weights thus providing a clear description about malicious and normalized images. This system model can be applied in real time for detecting diabetes from various ocular scans. Moreover, the system model will be integrated with the deep learning algorithm, and it is discussed in Section 3.

## 3. Proposed Solution Using Deep Learning Approach

We made a binary classifier to classify where the provided image of retina is malicious or normal. We experimented with deep learning algorithms and architecture to classify it. The below mentioned is the best result of classification we obtained through it. All the images in the dataset were resized to 244 × 244. Since the data size was low, we applied different techniques to increase the data size. We rescaled it to 1/255. We made rotation of image by 40° to increase the quantity of data. We shear the images by 02 value and zoomed it by 0.2 to increase the amount of dataset. Xception deep learning architecture was used for training purpose. Along with it, flatten layer was applied followed by two dense layers. The input size of Xception was 224 × 224 × 3. The flatten layer was applied to reduce its dimensions. The dense layer with 32 units was applied after it. The output layer was dense with sigmoid classifier and 1 unit. The model compiler with loss function was binary cross entropy and metric accuracy. The optimizer was RMSprop, and the classification accuracy for measurement can be represented in mathematical form as follows:

(7) $$\begin{array}{c} {\text{acc}}_{\text{clas}}=\sum_{i=1}^{n}\frac{t_{i}}{f_{i}}*100, \end{array}$$

where *t*_*i*_ and *f*_*i*_ represent the true and false values.

Equation (7) indicates that percentage of accuracy is measured by classification mechanism as distinct computational approaches are used. Thus, in deep learning, downward classified values can be calculated using the following equation:

(8) $$\begin{array}{c} {\text{down}}_{i}=\sum_{i=1}^{n}\mu_{i}*{\text{current}}_{\text{in}}, \end{array}$$

where *μ*_*i*_ denotes the learning rate and current_in_ represents the current generated values.

**Figure 2:**
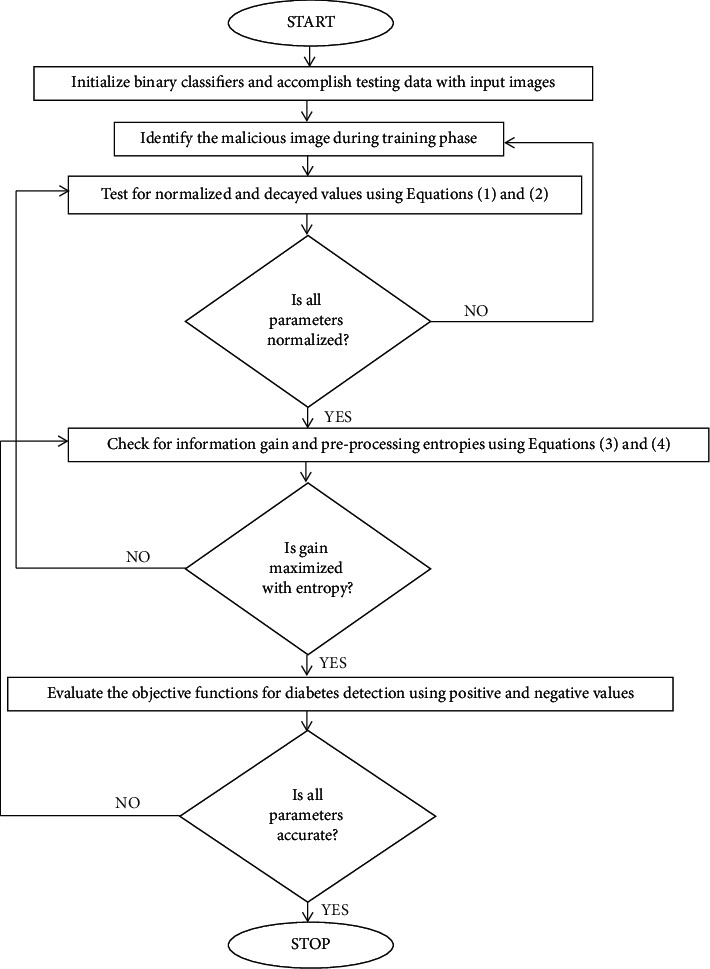
Flow chart of diabetes detection using deep learning.

The flow chart of integration with downward samples is deliberated in Figure 2 where all preprocessing entropies are checked with both before and after processing of images.

## 4. Results and Discussion

In binary classification task, our algorithms achieved a measurable success. We have created 2 classes, one for malicious and other nonmalicious. Training class consists of total 572 images, while validation class consists of total 220 images and testing class of 282 images. Despite the sensitivity, our algorithm has achieved the training accuracy of 96.68% and validation accuracy of 66.82% on applying attention layer in Xception along with dense neural network (DNN).

Firstly, we have performed with different pretrained models such as EfficientNetB0, EfficientNetB1, EfficientNetB2, EfficientNetB3, EfficientNetB4, EfficientNetB5, EfficientNetB6, and EfficientNetB7, and other models were used along with combination of dense layer. All the models showed the accuracy between 50 and 60%. Then, the Xception model was used till the last convolution layer. The last convolution layer was used as an input for fatten layer, followed by dense neural network (DNN) layers arrangement. The accuracy obtained for this architecture model at 10 epochs was 49 (training) and 50 (validation). Complete layer in Xception was used along with the dense neural network (DNN) layer arrangement used in previous experiment. Attention layer used Xception last layer as input, and it was input for DNN layer arrangement.

**Figure 3:**
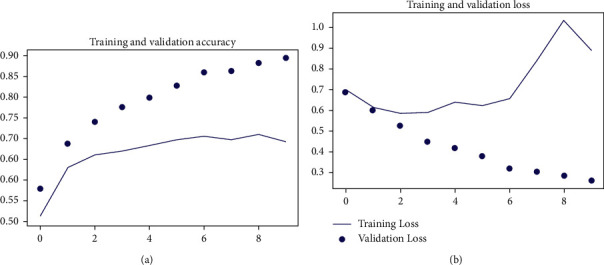
Training and validation accuracy for existing approach.

**Figure 4:**
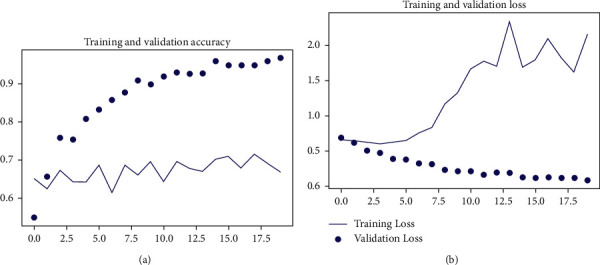
Training and validation accuracy of the proposed method.

The training accuracy obtained for this architecture model at 10 epochs was 89.34%, and validation accuracy for the same architecture model at 10 epochs was 69.09%. The training and validation accuracy and loss curve are shown in Figure 3. Complete layer in Xception was used along with the dense neural network (DNN) layer arrangement used in previous experiment. Attention layer uses Xception last layer as input, and it was input for DNN layer arrangement. 16 images batch was trained one each epoch, and weight was saved till 20 epochs. Following this process, the final training accuracy obtained was 96.68% and validation accuracy of 66.82% was obtained. The training and validation accuracy and loss curve are shown in Figure 4.

**Figure 5:**
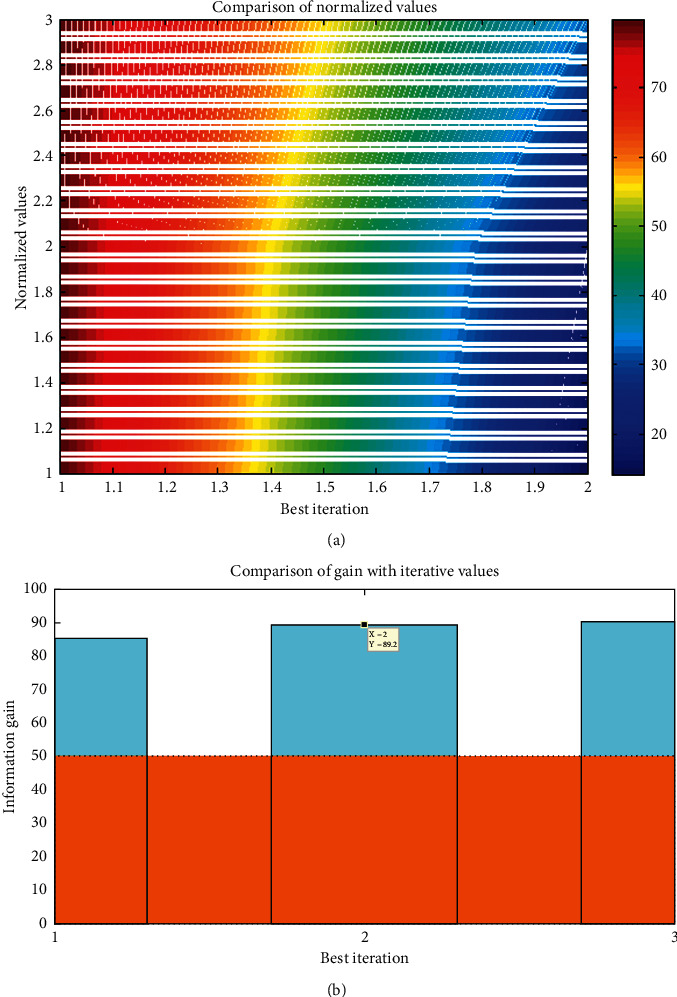
Convergence rate: (a) normalized values and (b) information gain.

From Figure 5, it can be observed that both normalized and information gain values are simulated and compared with conventional models [14, 15]. It is much clear that during the best iteration values which are observed at 80 points, the diabetes prediction is observed at clear stages and all images are normalized within certain intervals. In a similar manner, information gain is also achieved at best iteration values, and it is detected that matrix information gain for deep learning is attained at 89.2% which proves that data of diabetes are gained at extreme level values.

**Figure 6:**
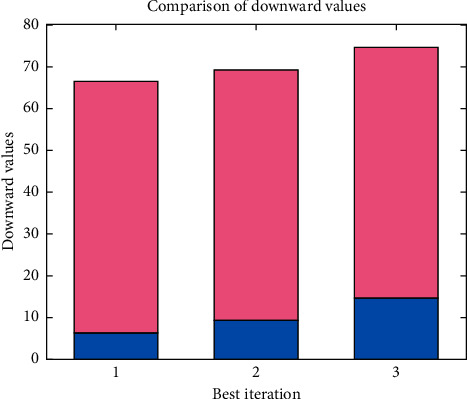
Downward values with iteration rate.

From Figure 6, it can be perceived that the best iteration rate is measured and downward rates for measured values are calculated. The stacked bar shows that learning rate is designed with current generated values where at initial stage it is much lesser but as iteration values are increased then maximum utilization of deep learning technique can be made probable with binary values. As a comparison result, the proposed method proves to be much effective with reduction of downward values as compared with existing models.

## 5. Conclusions

Diabetes retinopathy is incurable. To prevent vision loss, laser analysis is usually more effective when performed prior to retinal damage. If a person with diabetes receives regular eye care and treatment, whenever necessary, diabetic retinopathy will temporarily remove the blindness. This is very necessary as treatment in some cases does not even happen. However, implantation of retina images by ophthalmologists is very costly with the same automated systems that are much needed. Various algorithms are reviewed in this paper to automate the eye check-up and provide results that are closer to the standards. In this paper, we have presented a computational methodology for diabetic retinopathy with the help of deep learning. Throughout the task, it can be observed that deep learning is efficient for classification task and achieved measurable result. On applying certain deep learning algorithms, model results into the training accuracy of 96.68% and validation accuracy of 66.82%. Diabetic retinopathy can be considered as an effective and efficient method for diabetes detection.

### 5.1. Future Scope

Despite significant improvements, the expected increase in the number of diabetic retinopathy patients in the current times reminds us that more progress is yet to be made. Current research leads to a better understanding of molecular mechanisms, the development of new therapeutic targets, and the use of nanotechnology, coupled with improved diagnostic and cognitive technologies and collaborative health delivery systems, promise to enhance the ability to develop and maintain vision in diabetic patients.

## Figures and Tables

**Table 1:** Comparative analysis of approaches by other authors.

References	Approach	Database	Accuracy
[10]	Adaboost	DRIVE	95.97%
[11]	Improved matched filter	HRF	94.45%
[12]	Ensemble classifier	DRIVE/STARE	94.80%
[13]	IDP	Messidor	93.70%
[14]	Supervised classifier	Messidor	90.40

**Table 2:** Dataset folder description.

Testing	Malicious	141 images
	Normal	141 images

Training	Malicious	286 images
	Normal	286 images

Validation	Malicious	110 images
	Normal	110 images

## Data Availability

The datasets used and/or analyzed during the current study are available from the corresponding author on reasonable request.
